# Supplementary material for: Two zinc finger proteins with functions in m6A writing interact with HAKAI
Source: Nat Commun. 2022 Mar 2;13:1127. doi: 10.1038/s41467-022-28753-3 (PMC8891334; doi:10.1038/s41467-022-28753-3)
Supplement: Supplementary file 1 — Supplementary Information [file 41467_2022_28753_MOESM1_ESM.pdf]

Two zinc finger proteins with functions in m^{6}A writing interact with HAKAI

Zhang *et al.*

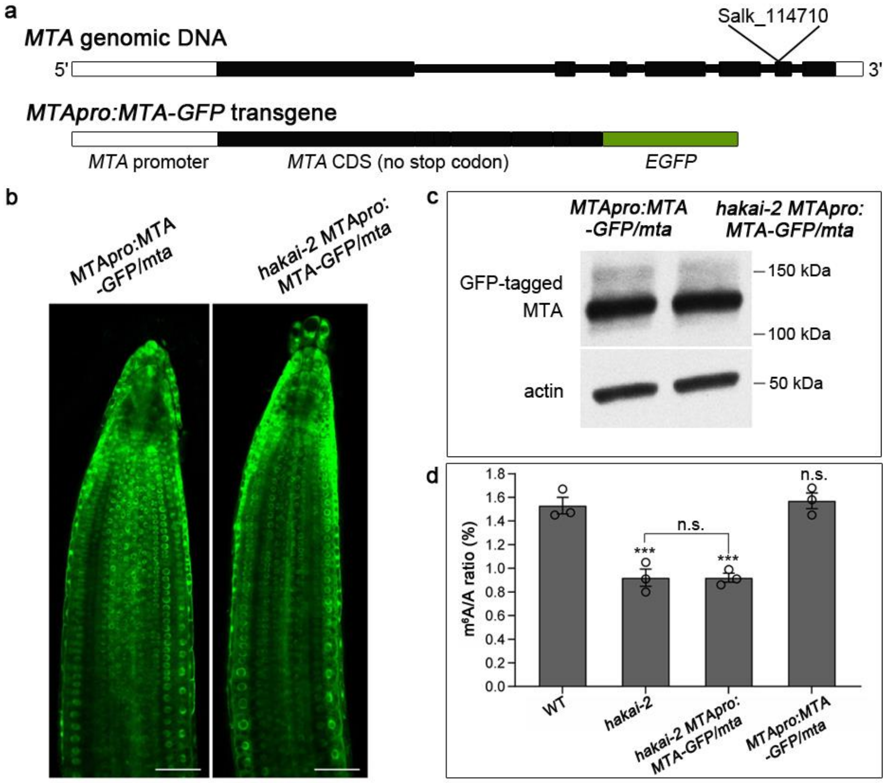

Supplementary Fig. 1. Generation and characterization of *hakai-2 MTApro:MTA-GFP/mta*. a Schematics of *MTA* genomic DNA and *MTApro:MTA-GFP* transgene for complementing Salk_114710. White rectangles denote UTRs of *MTA* genomic DNA, black rectangles denote exons and thick black lines represent introns. Salk_114710 is inserted in the 6th exon. CDS: coding sequence. EGFP: enhanced green fluorescent protein. **b** The localization of GFP-tagged MTA in primary root tips of 5-day old seedlings. Scale bar = 50 μm. Experiments in (**b**) were repeated independently at least three times, and representative images are shown. **c** GFP-tagged MTA protein levels checked by western blotting using an anti-GFP antibody. Experiments in (**c**) were repeated independently twice with similar results. **d** m^6A levels checked by two-dimensional thin layer chromatography (TLC) analysis. Data represent mean ± SE from 3 biological replicates and statistically significant differences relative to WT were analyzed by One-Way ANOVA (one-sided test) and marked with asterisks (***, $p < 0.001$; n.s., no significance). $p = 0.0006$ (*hakai-2*); $p = 0.0006$ (*hakai-2 MTApro:MTA-GFP/mta*). Source data are provided as a Source Data file.

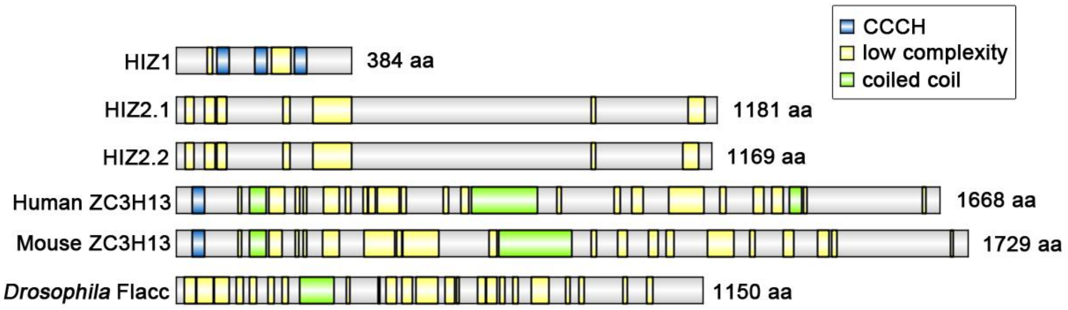

Supplementary Fig. 2. Protein domain analysis of *Arabidopsis* HIZ1 and HIZ2 and their homologs. HIZ2.1 and HIZ2.2 result from two splice variants of *HIZ2*. Protein domains were predicted via SMART (http://smart.embl.de/). Schematics of proteins were constructed by an online tool called Illustrator for Biological Sequences (IBS) (http://ibs.biocuckoo.org/online.php).

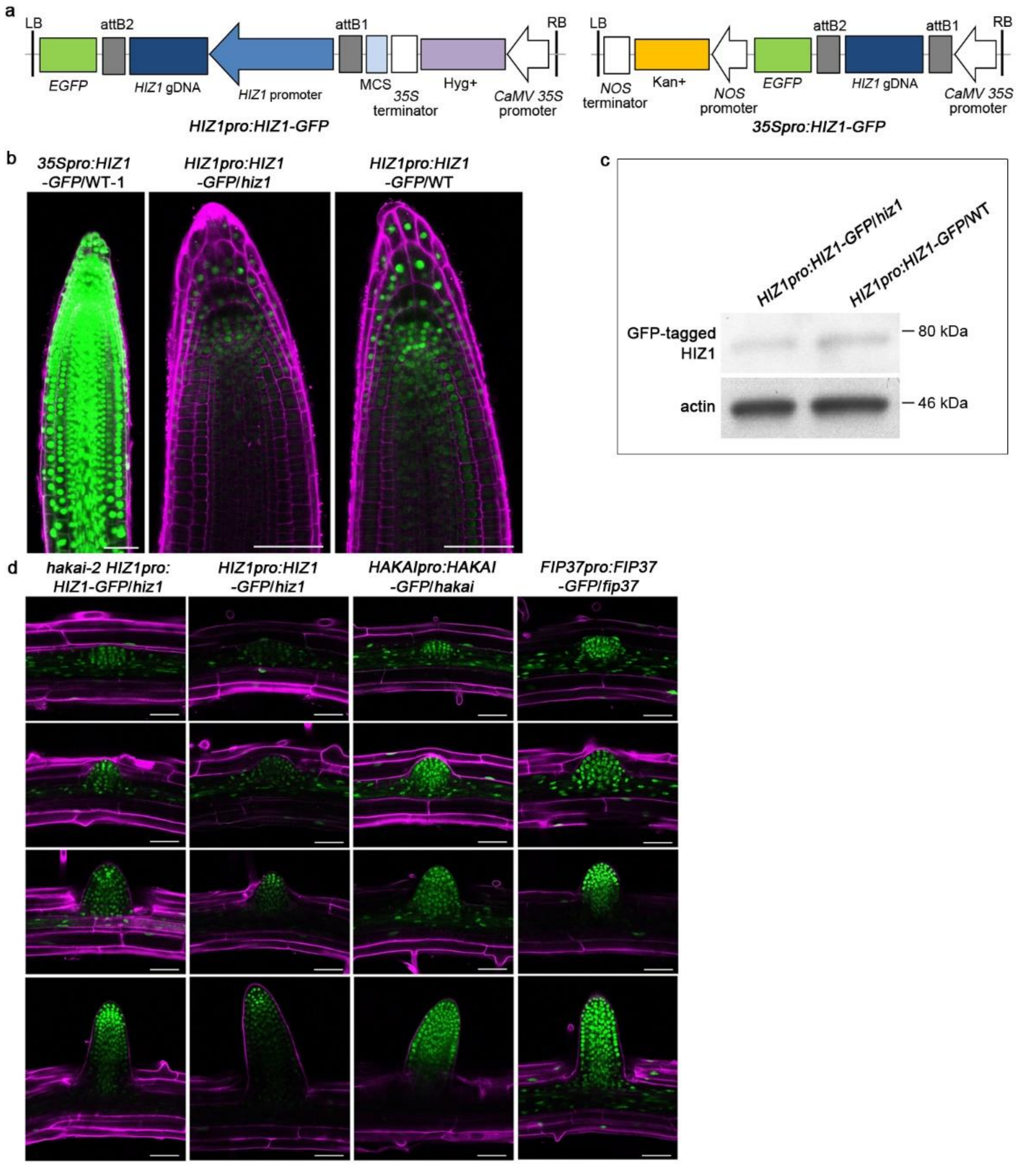

Supplementary Fig. 3. Localization and expression of different versions of GFP-tagged HIZ1. **a** Schematic of recombinant constructs containing *HIZ1*. *HIZ1* gDNA refers to *HIZ1* genomic DNA from start codon to nucleotides before the stop codon. Hyg^{+}: Hygromycin resistance for plants. Kan^{+}: kanamycin resistance for plants. MCS: multiple cloning site. **b** The expression of GFP-tagged HIZ1 in primary root tips of 3-day old seedlings. Scale bar = 50 μm. Experiments in (**b**) were repeated independently at least three times, and representative images are shown. **c** Western blotting demonstrating the protein levels of GFP-tagged HIZ1. Experiments in (**c**) were repeated independently three times with similar results. **d** Expression of GFP-tagged proteins in lateral root primordia (the first and second rows) and formed lateral roots (the third and fourth rows) of 9-day old seedlings. Scale bar = 50 μm. Experiments in (**d**) were repeated independently three times, and representative images are shown.

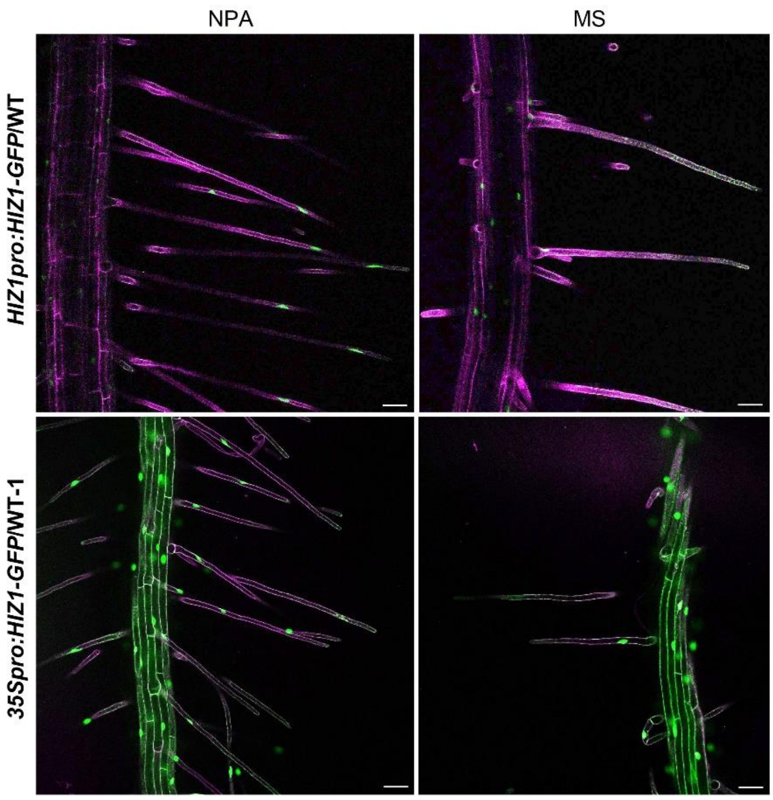

Supplementary Fig. 4. Expression of HIZ1-GFP in root hairs under the *N*-1-naphthylphthalamic acid (NPA) treatment compared with that on normal 0.5 × MS. Scale bar = 50 μm. Confocal images were taken from seedlings grown on 0.5 × MS with NPA (10 μM) or normal 0.5 × MS for 6 days after germinating on 0.5 × MS for 4 days. Experiments were repeated independently twice, and representative images are shown.

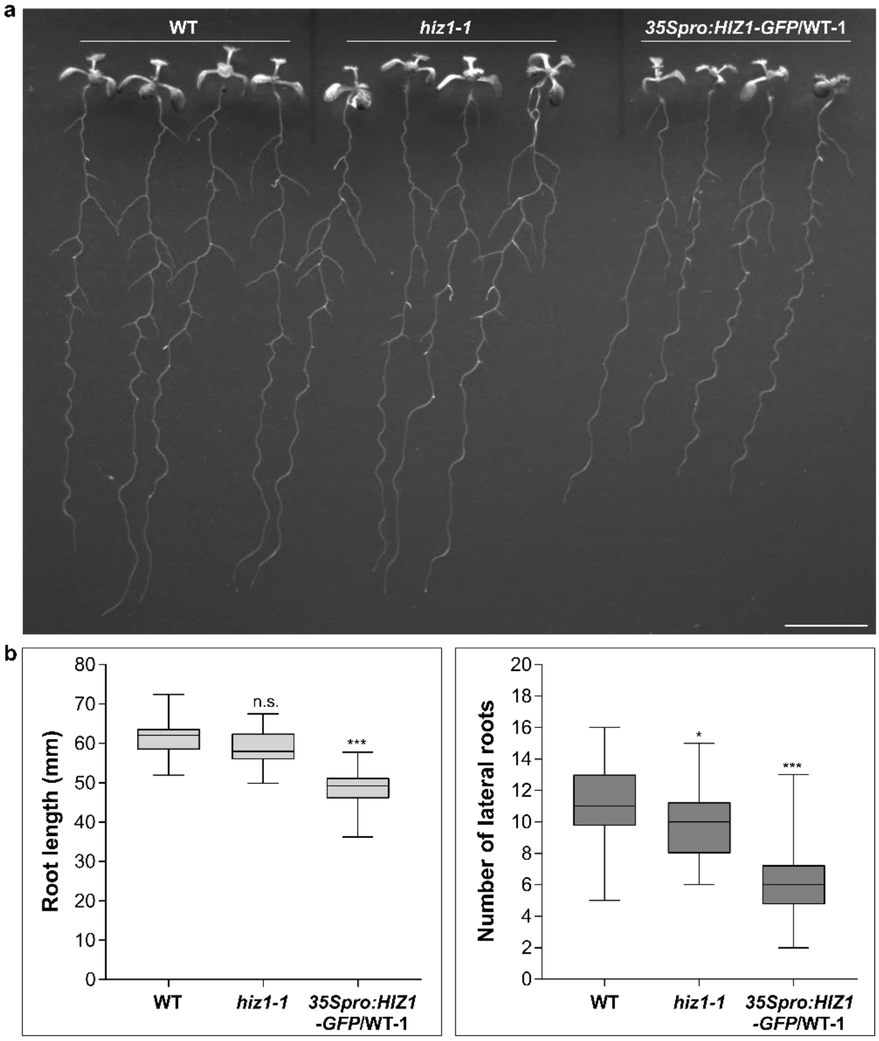

Supplementary Fig. 5. Root phenotypes of *hiz1* mutant and its overexpressor line *35Spro:HIZ1-GFP*/WT-1. a 10-day old *hiz1-1* and *35Spro:HIZ1-GFP*/WT-1 compared with WT. Seedlings were vertically cultured on 0.5 × MS plus 1% sucrose. Scale bar = 1 cm. **b** Statistical data showing root lengths and the number of lateral roots of seedlings in (**a**). Data represent mean ± SE (n = 34 individual seedlings) and statistically significant differences relative to WT were analyzed by One-Way ANOVA (one-sided test) and marked with asterisks (*, $p < 0.05$; ***, $p < 0.001$; n.s., no significance). In the data analysis for root length, $p = 0.054$ (*hiz1-1*) and $p < 0.0001$ (*35Spro:HIZ1-GFP*/WT-1); In the data analysis for number of lateral roots, $p = 0.0409$ (*hiz1-1*) and $p < 0.0001$ (*35Spro:HIZ1-GFP*/WT-1). In box plots, the center line in each box indicates the median. The lower and upper bounds of each box represent the first quartile (25%) and the third quartile (75%), respectively. The bottom and top of whiskers denote the minimum and maximum, respectively. Source data are provided as a Source Data file.

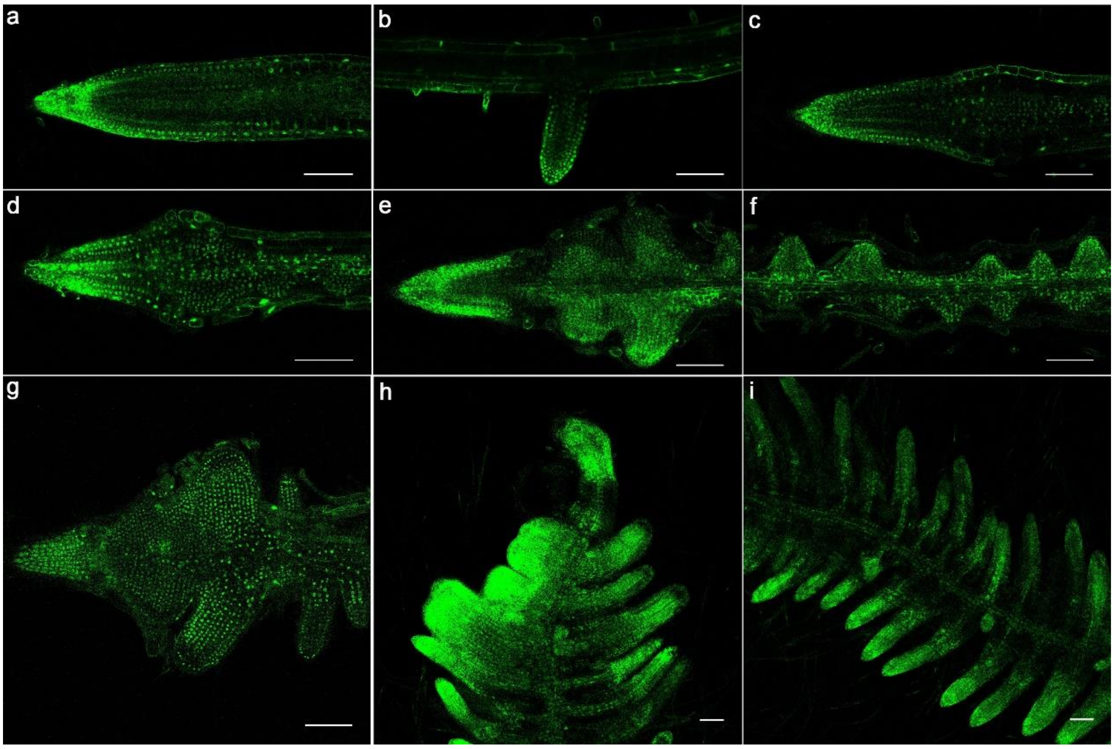

Supplementary Fig. 6. *MTApro:MTA-GFP/mta* roots under the confocal microscope after treatment with *N*-1-naphthylphthalamic acid (NPA) and then transferred onto 0.5 × MS with 1-naphthaleneacetic acid (NAA). a A primary root tip without treatments. **b** The elongation zone of a primary root without treatments. **c** A primary root tip induced by NAA for 1 day. **d** A primary root tip induced by NAA for 2 days. **e** A primary root tip induced by NAA for 3 days. **f** The elongation zone of a primary root induced by NAA for 3 days. **g** A primary root tip induced by NAA for 4 days. **h** A primary root tip induced by NAA for 5 days. **i** The elongation zone of a primary root induced by NAA for 5 days. In all images, scale bar = 100 μm. Experiments were repeated independently twice, and representative images are shown.

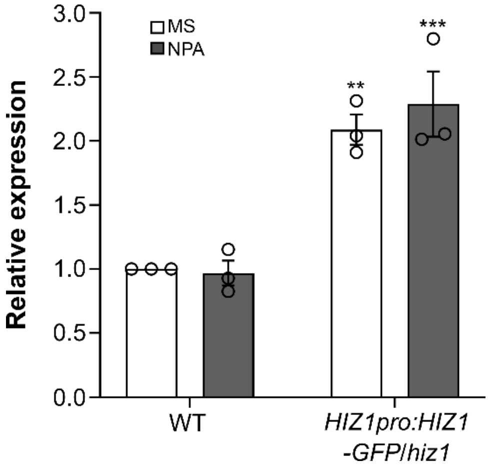

Supplementary Fig. 7. *HIZ1* transcript levels in *HIZ1pro:HIZ1-GFP/hiz1* compared with that in WT. RNA samples were prepared from seedlings grown on 0.5 × MS with *N*-1-naphthylphthalamic acid (NPA, 10 μM) or normal 0.5 × MS for 6 days after germinating on 0.5 × MS for 4 days. *CBP20* was used as a reference gene in RT-qPCR. Data represent mean ± SE from 3 biological replicates and statistically significant differences relative to WT were analyzed by Two-Way ANOVA (one-sided test) and marked with asterisks (**, $p < 0.01$; ***, $p < 0.001$). For *HIZ1pro:HIZ1-GFP/hiz1*, $p = 0.0017$ (MS) and $p = 0.0005$ (NPA). Source data are provided as a Source Data file.

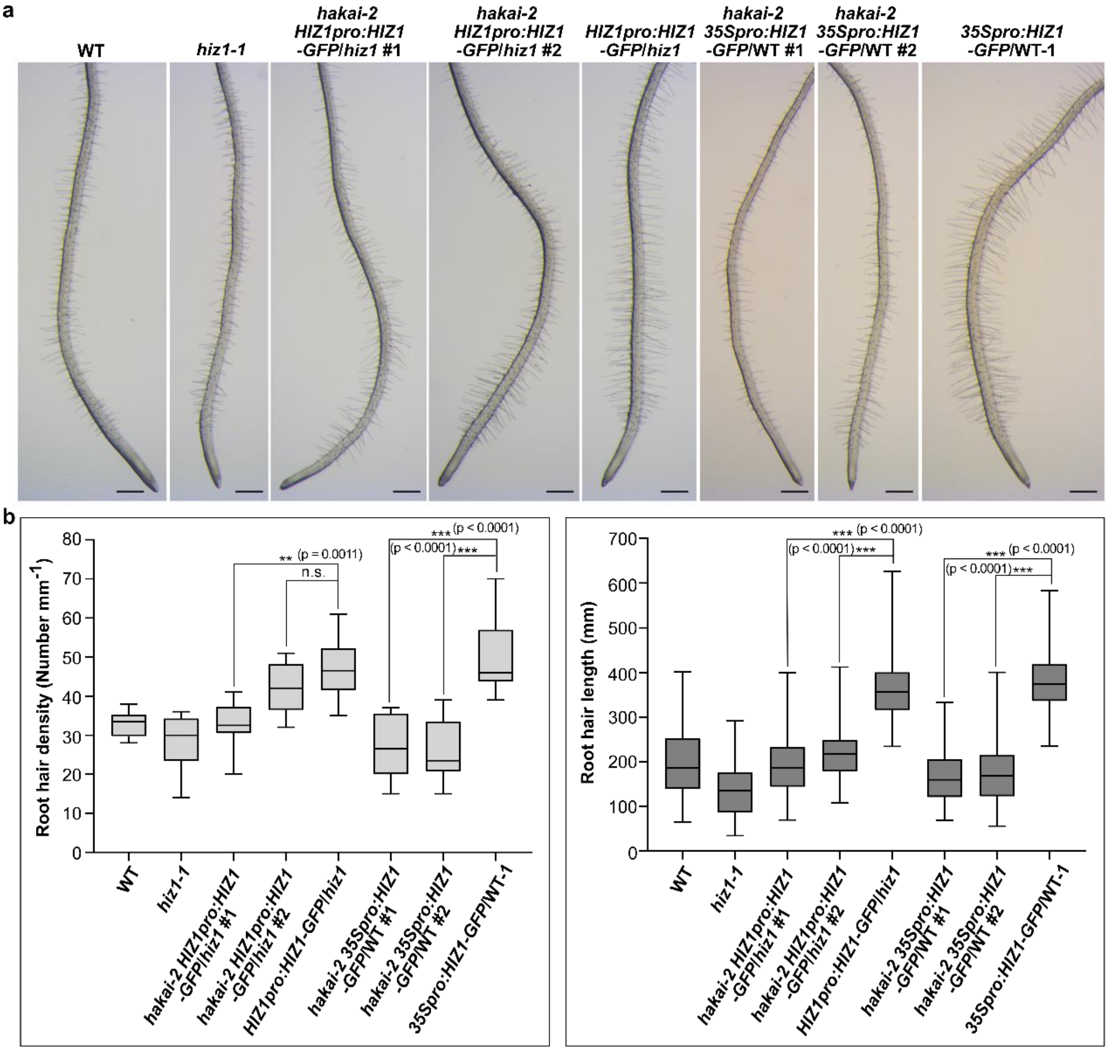

Supplementary Fig. 8. Root hair morphology of *HIZ1pro:HIZ1-GFP*/*hiz1* and *35Spro:HIZ1-GFP*/WT-1 in *hakai-2* background under the *N*-1-naphthylphthalamic acid (NPA) treatment. **a** Roots photographed under the Stereo Dissecting Microscope after being treated with NPA (10 µM) for 6 days. Scale bar = 500 µm. **b** Statistical data showing root hair density and lengths for lines in (**a**). The number of root hairs in the middle 1 mm segment of the imaged root represent the root hair density. The lengths of 10 root hairs on each side of the primary root in the middle 1 mm segment were measured to indicate its representative root hair length. 10 seedlings were used for the measurement for each line. Data represent mean ± SE and statistically significant differences were analyzed by One-Way ANOVA (one-sided test) and marked with asterisks (**, $p < 0.01$; ***, $p < 0.001$; n.s., no significance). In box plots, the center line in each box indicates the median. The lower and upper bounds of each box represent the first quartile (25%) and the third quartile (75%), respectively. The bottom and top of whiskers denote the minimum and maximum, respectively. Source data are provided as a Source Data file.

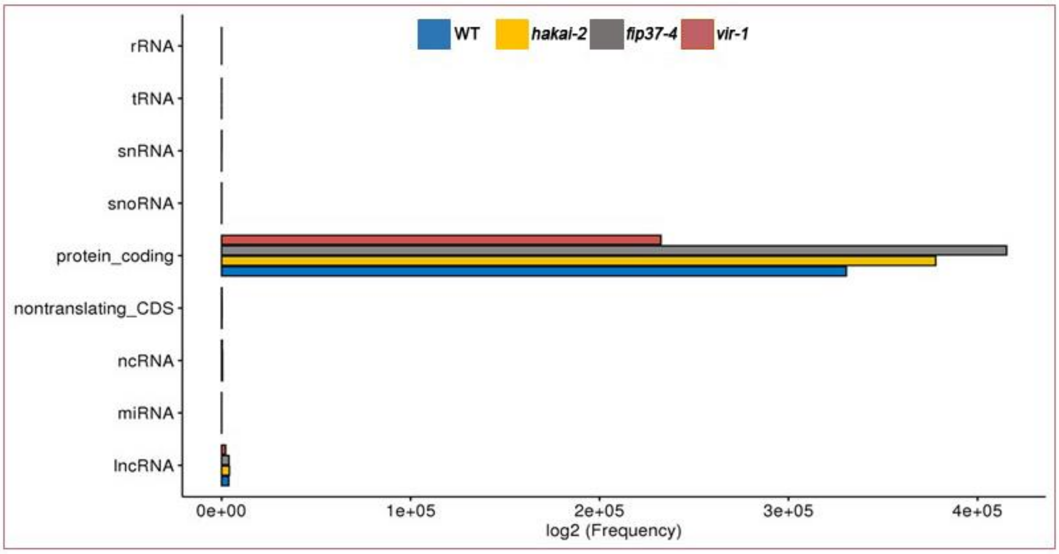

Supplementary Fig. 9. Gene type frequencies in WT, *hakai-2*, *fip37-4* and *vir-1*. rRNA: ribosomal RNA; tRNA : transfer RNA; snRNA: small nuclear RNA; snoRNA: small nucleolar RNA; CDS: coding sequence; ncRNA: non-coding RNA; miRNA: microRNA; lncRNA: long non-coding RNA.

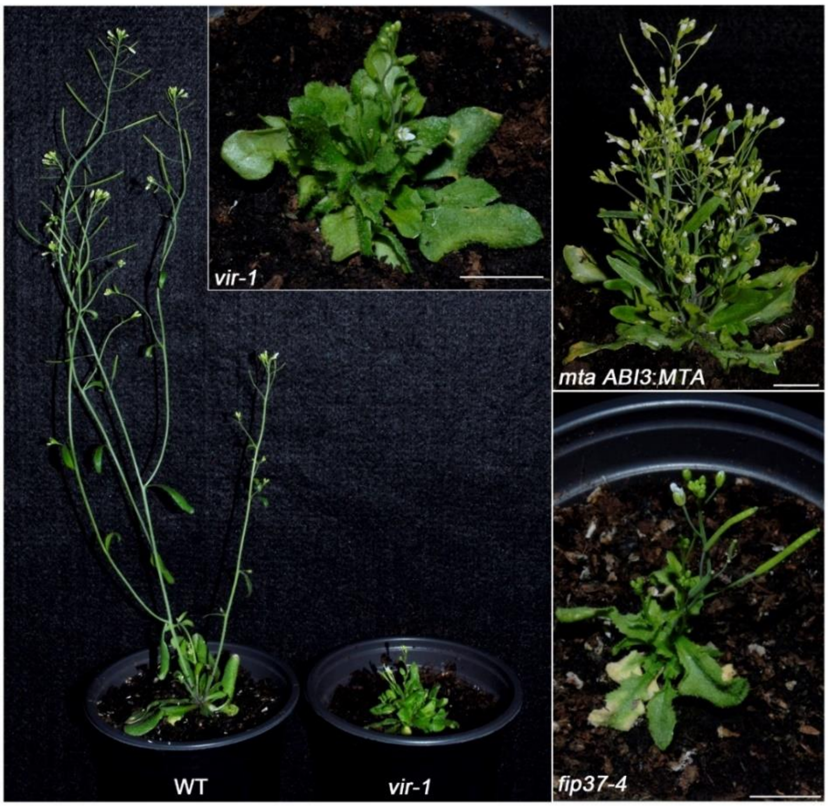

Supplementary Fig. 10. 5-week old hypomorphic mutants for core m^{6}A writer proteins and WT planted in compost. Scale bar = 1 cm.

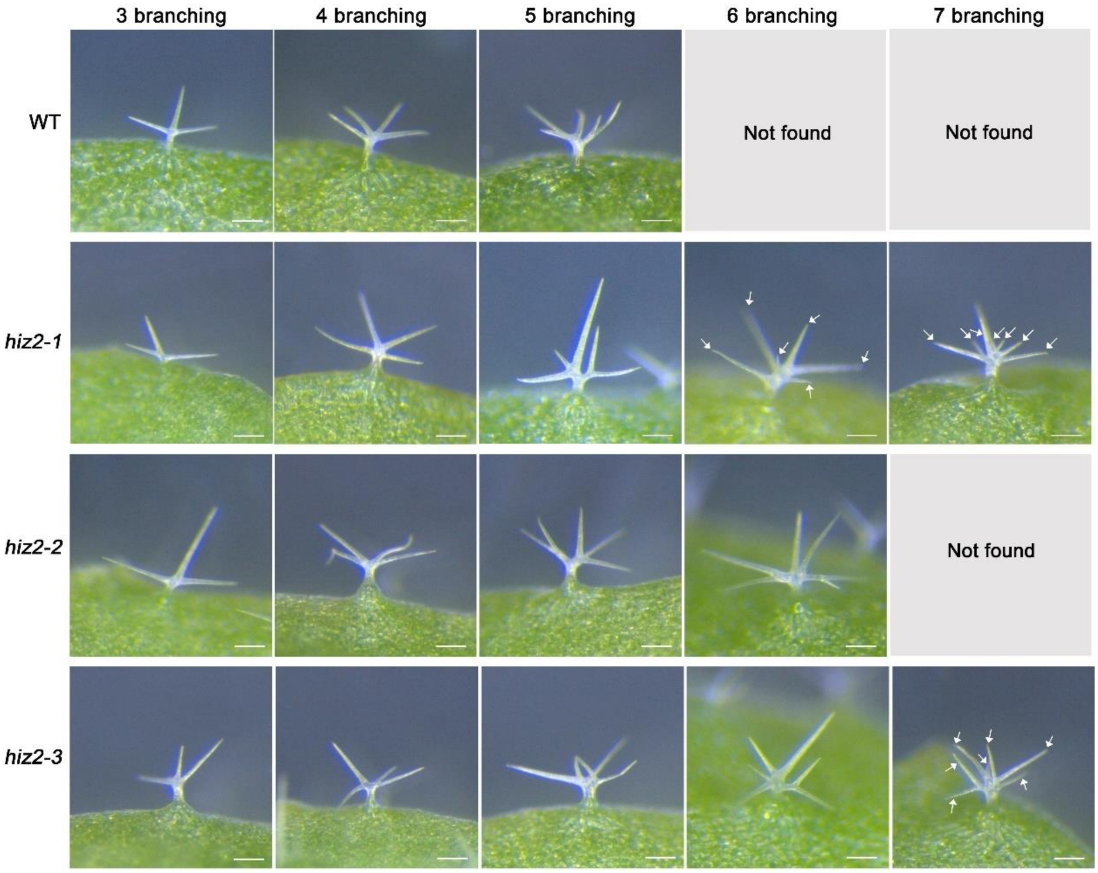

Supplementary Fig. 11. Representative images demonstrating different numbers of trichome branches on the first pair of rosette leaves from 2-week old plants cultured in compost. Scale bar = 100 μm.

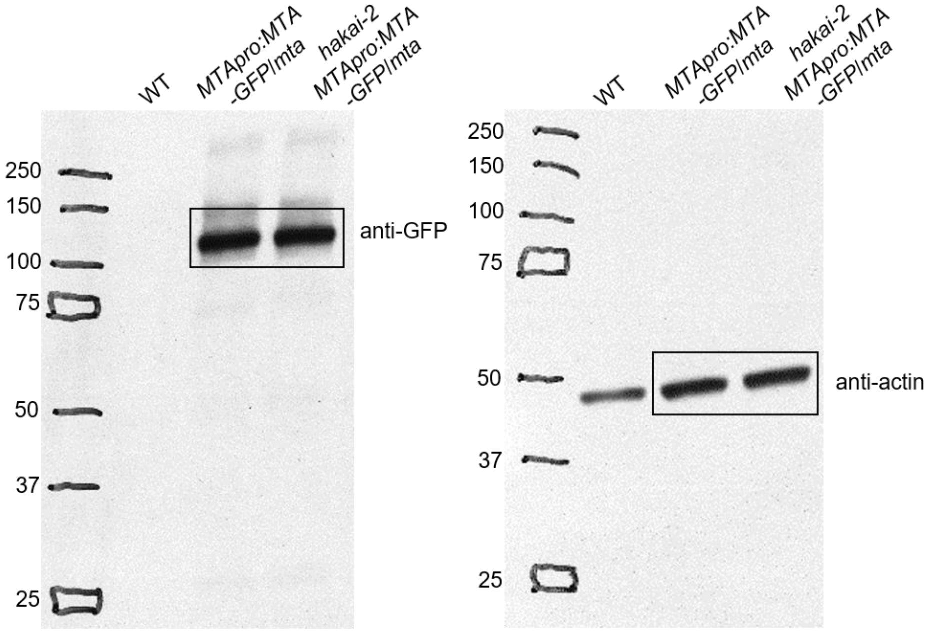

Supplementary Fig. 12. Uncropped scans of western blot membranes in Supplementary Fig. 1c. Cropped areas are labelled with rectangle squares.

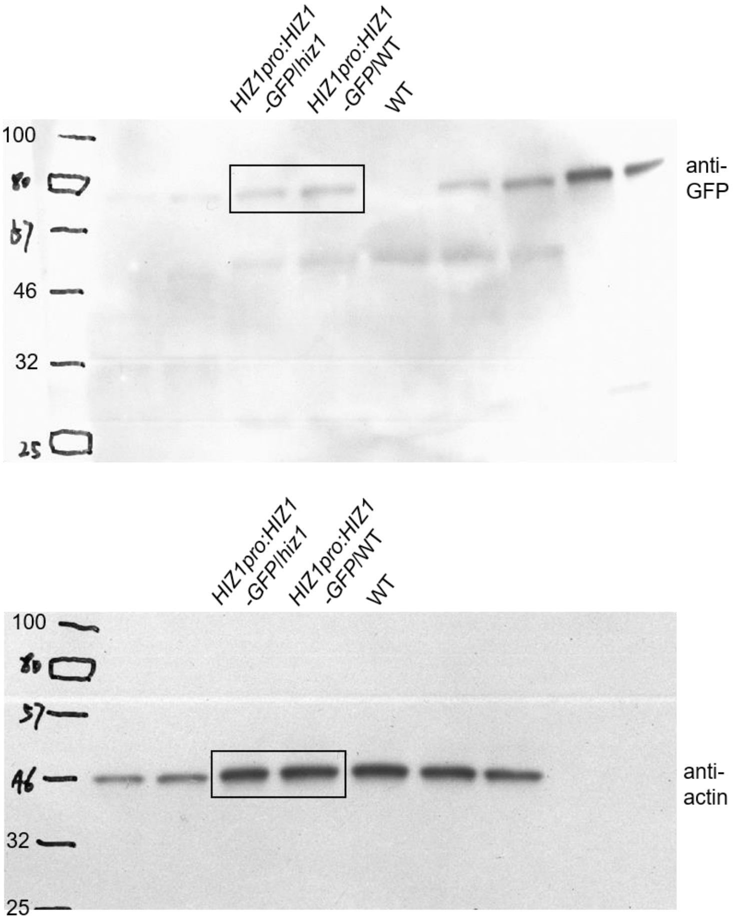

Supplementary Fig. 13. Uncropped scans of western blot membranes in Supplementary Fig. 3c. Cropped areas are labelled with rectangle squares.
