# Supplementary material for: Two zinc finger proteins with functions in m6A writing interact with HAKAI
Source: Nat Commun. 2022 Mar 2;13:1127. doi: 10.1038/s41467-022-28753-3 (PMC8891334; doi:10.1038/s41467-022-28753-3)
Supplement: Supplementary file 3 — Description of Additional Supplementary Files [file 41467_2022_28753_MOESM3_ESM.pdf]

Description of Additional Supplementary Files

Supplementary Data 1 Pull-down proteins with GFP-tagged HAKAI or MTA.

Supplementary Data 2 List of peaks that disappear or decrease in the writer complex member mutant *hakai-2*.

Supplementary Data 3 List of peaks that disappear or decrease in the writer complex member mutant *fip37-4*.

Supplementary Data 4 List of peaks that disappear or decrease in the writer complex member mutant *vir-1*.

Supplementary Data 5 Gene Ontology analysis of the writer complex member mutants.

Supplementary Data 6 List of differentially expressed genes in *vir-1*, and the distribution of missing m^6A peaks in *vir-1* among *vir-1* up and down regulated transcripts.

Supplementary Data 7 List of methylated ARF7 regulated transcripts, and a list of ARF7 regulated transcripts that lose m^6A in *vir-1*.

Supplementary Data 8 List of WT methylated, differentially expressed root hair transcripts.

Supplementary Data 9 Primers used in this study.
